# Primary upper lumbar hernia repaired by transabdominal preperitoneal approach technique using a self-expanding mesh with a memory-recoil ring, report of a case

**DOI:** 10.1186/s40792-022-01564-w

**Published:** 2023-01-02

**Authors:** Reika Yamashita, Katsuhito Suwa, Tomoyoshi Okamoto, Ken Eto

**Affiliations:** 1 Department of Surgery, The Jikei University Daisan Hospital, Komae-shi, Tokyo, Japan; 2 Department of Digestive Surgery, The Jikei University Hospital, Komae-shi, Tokyo, Japan

**Keywords:** Self-expanding mesh, Transabdominal preperitoneal repair (TAPP), Upper lumber hernia

## Abstract

**Background:**

Upper lumber hernia is a rare entity which can cause obstruction and strangulation. Laparoscopic technique has been considered effective for such hernia repairs; however, there is no report of use of the self-expanding mesh.

**Case presentation:**

A 77-year-old woman visited to our hospital complaining of a bulge of about 5 cm in the left lumbar dorsal region while standing. Abdominal CT and MRI scans showed a fascial defect in the left lumbar abdominal wall and confirmed the presence of a hernia, in which retroperitoneal fatty tissue and the descending colon protruded. Transabdominal preperitoneal repair (TAPP) was performed and the operative findings revealed the hernia orifice, 3 × 2.5 cm in diameter, between two intercostal nerves. To avoid nerve injury or entrapment, the number of mesh fixation was desirable minimum; therefore, a self-expanding mesh with a memory-recoil ring was used. The mesh, 9.5 × 13 cm in diameter, was placed and tacked to the abdominal wall at two points, 1 cm ventral and dorsal to the hernia orifice. The postoperative course was uneventful and no pain or recurrence was observed with follow-up of 6 months.

**Conclusion:**

We herein present a case of upper lumber hernia successfully repaired by TAPP with a self-expanding mesh.

## Background

Upper lumbar hernia is a rare disease that occurs in the upper hip triangle (Grynfeltt-Lesshaft's triangle) surrounded by the 12th subcostal, the posterior border of the internal oblique muscle, the inferior posterior serratus muscle, and erector spinae [1–3]. Upper lumbar hernias are often adapted for surgery due to the relatively high risk of incarceration and mesh repair is recommended in terms of recurrence. Recently, there have been several reports of transabdominal preperitoneal repair (TAPP) for lumbar hernia [2, 5–12]. Anatomically, upper lumbar herniation requires sufficient attention for mesh fixation due to the adjacent intercostal nerves. Self-expanding mesh patch frequently used for inguinal and ventral hernia repairs requires no or less fixation and is suitable for hernias with such situation. A search of PubMed reveals no report of laparoscopic upper lumbar hernia repair using a self-expanding mesh.

## Presentation of case

**Fig. 1:**
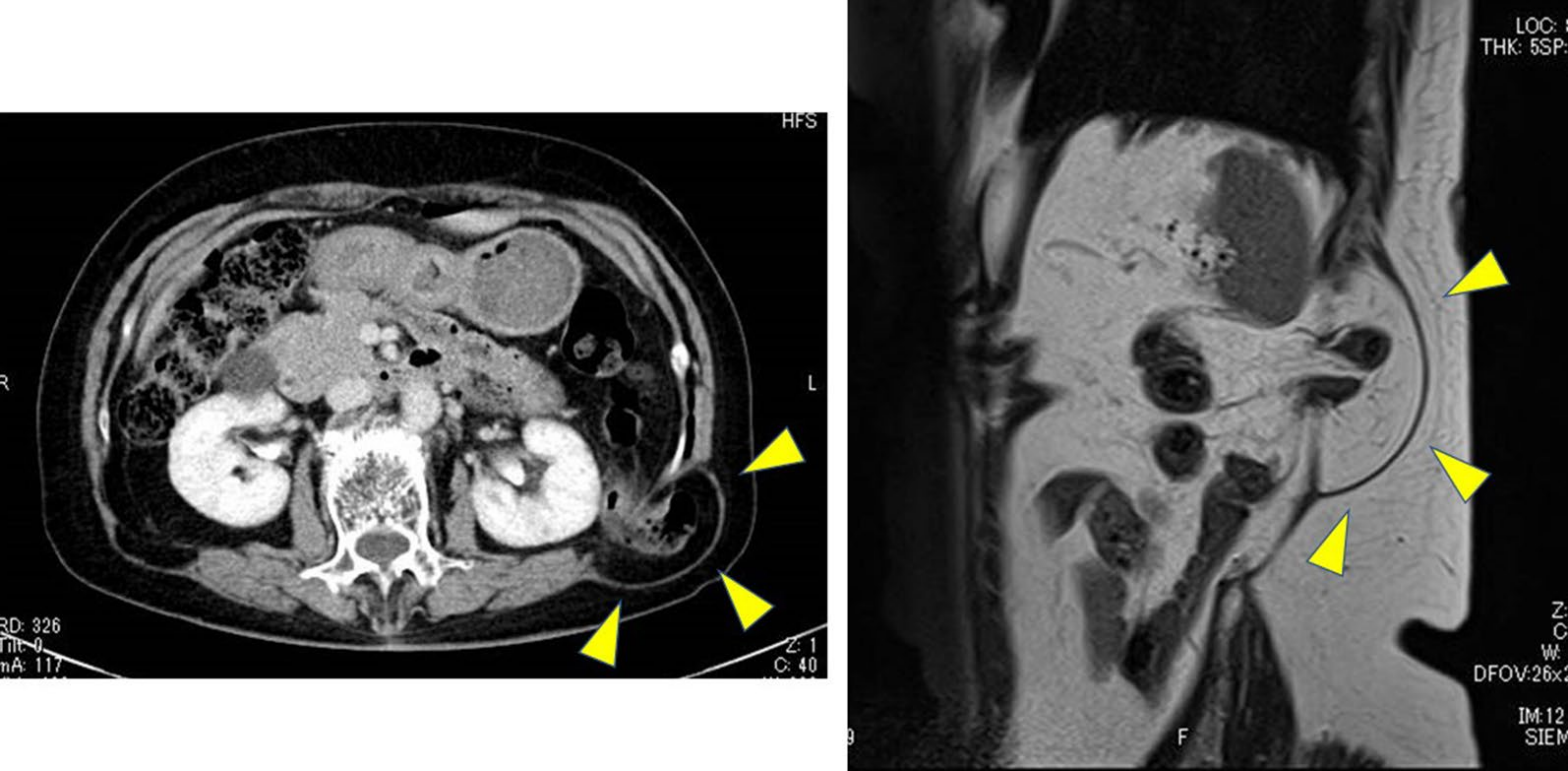
Abdominal CT and MRI. The left: Preoperative computed tomography image. The right: preoperative magnetic resonance image. A fascial defect was in the left lumbar abdominal wall and in which retroperitoneal fatty tissue and the descending colon protruded (arrows)

The patient, a 77-year-old female, body mass index 20.5 kg/m^2^, presented at our department complaining of hen-egg-sized swelling in the left upper lumbar region with duration of a year. Physical examinations confirmed a reducible hen-egg-sized bulge on her left upper back and CT scan showed a fascial defect in the left lumbar abdominal wall, through which the retroperitoneal fatty tissue and the descending colon protruded to the subcutaneous layer (Fig. 1). At the time of initial examination, there was no specific sign or symptom of bowel obstruction. She underwent laparoscopic sacrocolpopexy for pelvic organ prolapse 6 months prior to visiting our clinic but had no history of trauma, infection, weight loss, or surgery in that area. She has no history of either intraperitoneal or preperitoneal cavity surgery. The diagnosis of left superior lumbar hernia was entertained and an elective laparoscopic operation through a retroperitoneal approach was performed.

## Operative technique

**Fig. 2:**
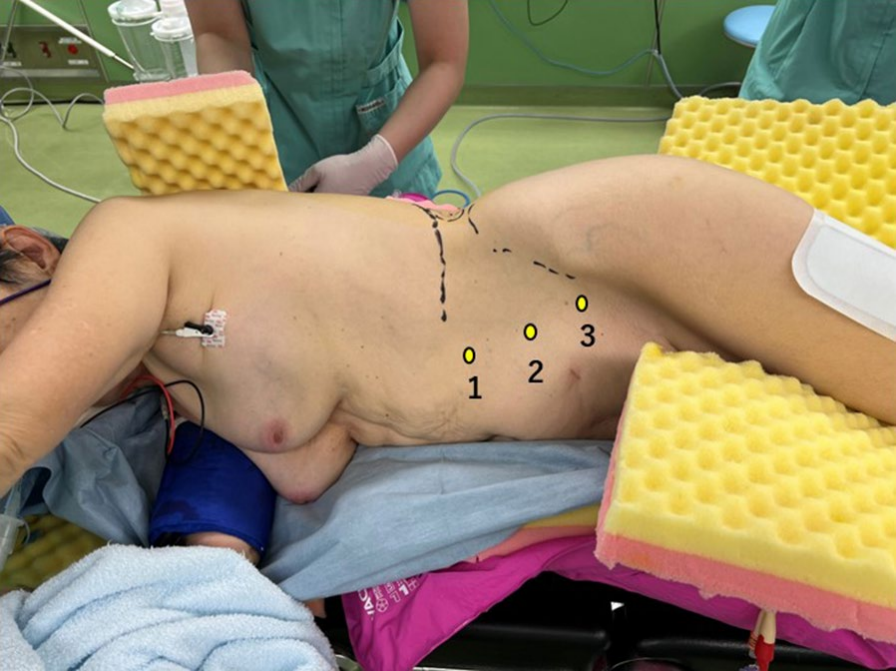
Operative posture of the patient. Under endotracheal general anesthesia, the patient was placed in the right lateral position. The trocar sites were indicated as 1, 2 and 3

**Fig. 3:**
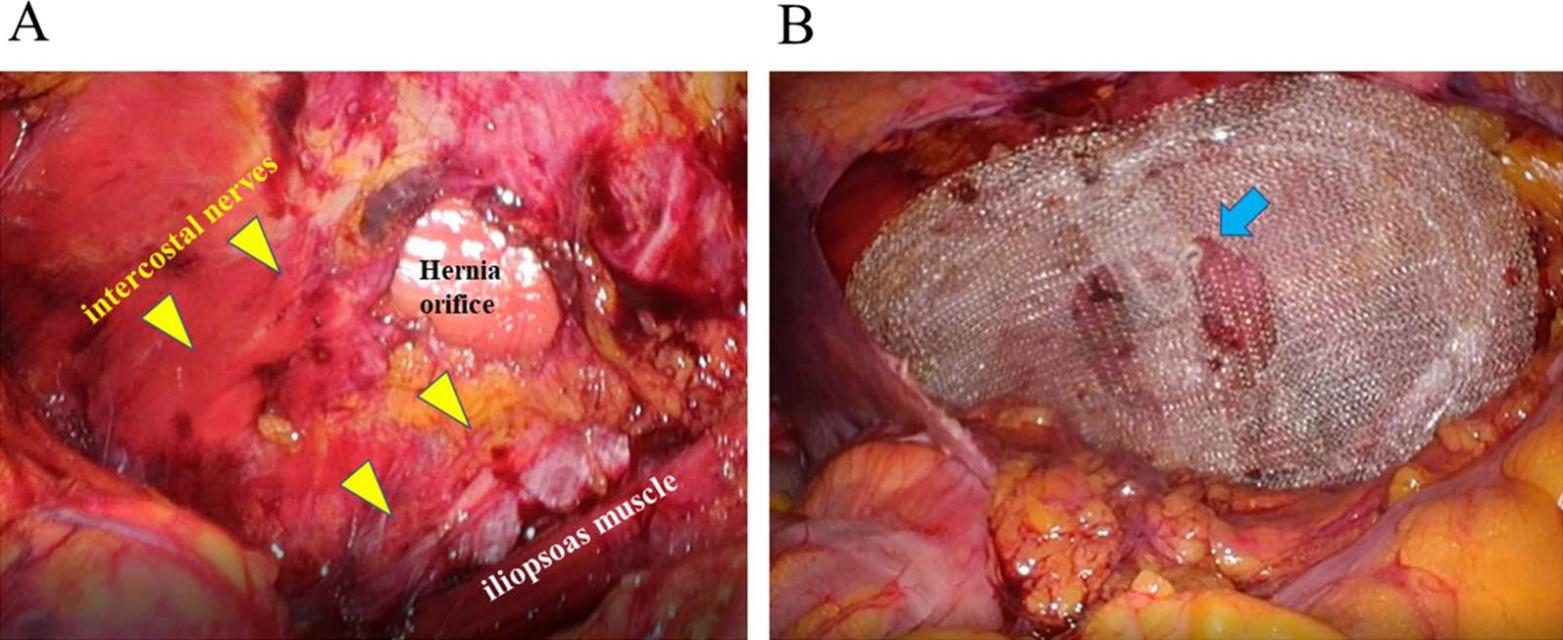
Intraoperative view. **A** Hernia orifice. The hernia orifice, 3 × 2.5 cm in diameter, was identified between two intercostal nerves (yellow arrows). **B** Placement of the self-expanding patch mesh. A modified Kugel patch was placed as its center (blue arrow) was positioned just above the hernia orifice and tacked to the abdominal wall at two points, 1 cm ventral and dorsal to the orifice taking care not to damage the nerves

Under endotracheal general anesthesia, the patient was placed in the right lateral position (Fig. 2) and prepared. Through a small skin incision was made on the left upper quadrant, the abdominal cavity was entered and then a 12 mm trocar was placed. Additional two 5-mm trocars were placed into the left middle abdomen. A transverse incision in the peritoneum approximately 5 cm ventral to the paracolic fossa of the descending colon, and the retroperitoneal space was dissected dorsally. The hernia sac was bluntly dissected from the pseudo-sac and reduced to the intraabdominal side. The anterior surface of the iliopsoas muscle was sufficiently dissected for subsequent mesh placement. The hernia orifice, 3 × 2.5 cm in diameter, was identified between two intercostal nerves (Fig. 3a). A modified Kugel patch (BD-BARD, Warwick, RI, USA), 9.5 × 13 cm in diameter, was placed over the hernia orifice and tacked to the abdominal wall at two points, 1 cm ventral and dorsal to the orifice taking care not to damage the nerves (Fig. 3b).

## Postoperative course

The postoperative course was uneventful and the patient was discharged on the sixth postoperative day. No pain or recurrence was encountered with follow-up of 6 months.

## Discussion

Lumbar hernias are quite as compared to other ventral abdominal wall hernias. According to the anatomical location of the defect, lumber hernias are divided into Grynfeltt hernia (the superior triangle) and Petit hernia (the inferior triangle) [13–17]. The superior lumber triangle is formed by the 12th rib and the serratus posterior inferior muscle. The inferior lumber triangle is formed by the iliac crests. Superior lumbar hernia accounts for the most [1] because anatomically superior lumbar triangle is probably larger and weaker than inferior triangle.

They are classified as either congenital or acquired. Congenital lumbar hernias represent 20%, are usually seen infancy. Acquired lumbar hernias are primary or spontaneous and secondary. Acquired lumbar hernias can be further classified as either primary or secondary. Most of them are primary and precipitated by conditions associated with aging, increased intra-abdominal pressure, chronic bronchitis and extreme thinness, etc. [17, 18]. Secondary type lumbar hernias are often associated with surgical incisions (retroperitoneal operations and harvesting a bone graft from the iliac crest), trauma or lumbar abscess [6, 19, 21]. This case was a primary lumbar hernia.

The most common symptom is a posterior and regional protruding bulge in the lumbar region. Symptoms are variable and may be asymptomatic, associated with local and abdominal discomfort, or marked local tenderness. Tenderness is caused by distribution of the sciatic nerve or bowel obstruction due to intestinal fits [5, 7, 10].

The best treatment option for lumbar hernia is a surgical repair because hernia orifice gradually is enlarged and become difficult to repair. Sometimes, intestine can be strangulated in hernia orifice, intestinal tract necrosis and patient's condition worsens.

Surgery is the most common treatment for lumber hernia. Several surgical methods have been applied. For example, primary suture closure of the hernia orifice and reinforce the oblique muscle group and dorsal muscle group (Petit surgery) [6, 12], bone transfer, various soft tissue flaps, and mesh repair. Tension-free repair using prosthetic meshes has been increasing, and recently, laparoscopic repair cases have also been common as minimally invasive surgery [5–12]. There are many benefits of the laparoscopic repair; ① an excellent anatomic view of the whole of the lumbar area, hernia type and content, and the edges of the fascial defect, ② the hernia orifice and the surrounding potentially vulnerable abdominal wall can be covered with a mesh, ③ even highly obese patients can be operated with a small incision, and ④ less postoperative pain, wound infection, and shorter hospitalization period [7, 22].

As shown in Table 1, in the previous reports of laparoscopic repair for the upper lumbar hernia, 7 cases used composite mesh out of 27 cases, 14 cases used polypropylene mesh, 1 case used expanded polytetrafluoroethylene mesh, 3 cases of unknown details, and 1 case used self-gripping mesh. There were 17 cases where mesh was fixed with tackers, 3 cases with suturing, 2 cases with stapler, and 2 cases where the mesh was only placed without fixation.

**Table 1:** Reported cases of laparoscopic repair for the upper lumbar hernia

No	Author	Year of publication	Age	Sex	Complaints	Laparoscopic/Open	Location	Hernia content	Hernia orfice size (cm)	Jia	Mesh size (cm)	Mesh fixation	fixed number	Operating time	Hospital stay(days)
1	Habib E [23]	2002	65	M	Swelling	Laparoscopic	Right	Retroperitoneal Fat	3 × 4	Prolene	13 × 13	Tacker	> 10	ND	ND
2	Meinke AK [9]	2003	78	M	Pain	Laparoscopic	ND	Fat And Cecal	Unknown	Unknown	Unknown	ND	ND	ND	ND
3	Maeda K [24]	2003	74	F	Swelling	Laparoscopic	Left	Descending Colon	2.5	Prolene	6 × 6	Suture	ND	ND	ND
4	Ipek T [25]	2005	36	F	Pain, swelling	Laparoscopic	Left	None	8 × 10	PTFE	11 X 13	Suture	> 10	ND	2
5	Iannitti DA [26]	2007	36	M	Swelling	Laparoscopic	Right	Small And Large Bowel	2 × 3	Composite	14 × 17	Tacker	> 10	ND	ND
6	Yavus N [22]	2009	51	F	Pain	Laparoscopic	Left	Descending Colon	6 × 8	Prolene	10 × 15	Anchor	> 10	120	10
7		2009	39	M	Pain, swelling	Laparoscopic	Left	Omentum	4 × 6	Prolene	10 × 15	Tacker	> 10	75	6
8		2009	81	F	Pain	Laparoscopic	Right	Omentum	8 × 10	Prolene	10 × 20	Tacker	> 10	150	13
9		2009	48	F	Pain, swelling	Laparoscopic	Left	Omentum	4 × 6	Composite	10 × 15	Tacker	> 10	90	7
10		2009	46	F	Pain, swelling	Laparoscopic	Left	Omentum	6 × 10	Composite	10 × 20	Tacker	> 10	90	11
11		2009	45	F	Pain	Laparoscopic	Right	Omentum	4 × 6	Prolene	10 × 15	Tacker	> 10	120	7
12		2009	31	M	Pain, swelling	Laparoscopic	Left	Descending Colon	6 × 10	Prolene	10 × 15	Tacker	> 10	90	3
13		2009	75	F	Pain, swelling	Laparoscopic	Right	Ascending Colon	Unknown	Composite	8 × 15	ND	ND	ND	ND
14	Lim MS [27]	2011	76	F	Pain, swelling	Laparoscopic	Left	Descending Colon	6 × 5	Prolene	15 × 15	Tacker	ND	ND	5
15	Links DJ [19]	2011	40	M	Pain	Laparoscopic	Left	Sigmoid Colon	Unknown	Composite	16 × 21	Tacker	ND	ND	ND
16	Nam SY [28]	2011	70	F	Pain, swelling	Laparoscopic	Left	Retroperitoneal Fat	1.5 × 1.5	Prolene	15 × 15	Tacker	> 10	ND	5
17	Tobias-Machado M [8]	2012	62	F	Swelling	Laparoscopic	Left	Left Colon	8 × 8	Prolene	13 × 13	Staple	> 10	100	2
18	Varban O. [29]	2013	61	F	Pain, swelling	Laparoscopic	Left	Colon	5 × 7	Prolene	20 × 25	Tacker	> 10	110	3
19	Suarez S [30]	2013	51	F	Pain, swelling	Laparoscopic	Right	Omentum	Unknown	Unknown	Unknown	Tacker	ND	ND	ND
20	Wei CT [31]	2014	76	M	Swelling	Laparoscopic (SILS)	Left	Abdominal Fat	3 × 3	Prolene	13 × 13	Autosuture stapler	> 10	ND	4
21	Walgamage TB [4]	2015	33	M	Swelling	Laparoscopic	Right	Retroperitoneal Fat	5 × 5	Vypro	9 × 15	Tacker	> 10	ND	ND
22	Matsuda A [5]	2016	49	M	Swelling	Laparoscopic	Left	Descending Colon	5 × 8	Composite	14 × 18	Tacker	> 10	ND	ND
23	Kaminski S [32]	2017	43	M	Pain	Laparoscopic	Right	Ascending Colon And Cecum	Unknown	Unknown	orifice + 4 cm	only placement	none	ND	ND
24	Sarwal A [33]	2018	65	F	Swelling	Laparoscopic	Left	None	3 × 3	Prolene	15 × 15	Tacker	ND	ND	2
25	Moriyama H [34]	2020	72	F	Swelling	Laparoscopic	Left	Left Colon	Unknown	Composite	20 × 25	Tacker	ND	ND	ND
26	Nakahara Y [35]	2020	65	F	Swelling	Laparoscopic	Left	Retroperitoneal Fat	2 × 1.5	Self-fixating mesh	10 × 15	None	none	50	5
27	Kiam JS [36]	2021	66	F	Pain, swelling	Laparoscopic	Right	Fat And Ascending Colon	4 × 8	Prolene	Unknown	Suture	> 10	ND	ND
28	Our case		77	F	Swelling	Laparoscopic	Left	Descending Colon	3 × 2.5	None	9.5 × 13	Tacker	2	90	6

*ND* not described

The upper lumbar hernia is anatomically surrounded by the intercostal nerves. The intercostal nerves dominate the skeletal muscles that form the chest and abdominal wall and the skin of the chest and abdominal wall (anterior and lateral). They function not only as motor nerves for breathing, raising the arm, and flexing the upper body but also as sensory nerves for the body surface. Accidental entrapment of nerves can cause postoperative pain and should be avoided. A self-expanding mesh can be easily unrolled and fixed to the abdominal wall due to Pascal’s law in the preperitoneal space and thus requires no or less fixation [37]. In terms of complications associated with nerve, we adopted this type mesh for laparoscopic lumbar hernia repair. The mesh was fixed in only two points to avoid injury of intercostal nerves running adjacent hernia. Postoperative pain was minimum, and no recurrence was observed with follow-up of 6 months.

Similarly, self-gripping mesh is a good option in that it requires little or no fixing. However, self-grip mesh can be difficult to deploy in the body cavity. In rare cases such as lumber hernias, the mesh with a memory ring is easier to use, because it is self-expanding and easy for anyone to use.

One limitation of using a self-expanding mesh is that there are a very few variations in size. The maximum size of the mesh used in Japan is 9.5 × 13.0 cm. Considering sufficient overlap of this mesh, it should be used for a hernia with diameter of less than 5 cm.

Concerns about mesh with non-absorbable memory-recoil ring may exist in regard to long-term safety. Some authors reported bowel complications related to self-expanding mesh in intraperitoneal only mesh ventral hernia repair; however, in extraperitoneal hernia repair, there have been no reports demonstrating complications due to ring breakage.

## Conclusion

TAPP using a mesh with a self-expanding mesh seems to be useful for upper lumbar hernia repair.

## Data Availability

All data generated or analyzed during this study are included in this published article.

## Abbreviation

TAPP: Transabdominal preperitoneal repair
